# The effect of *MDR1 C3435T* polymorphism on the eradication rate of *H. pylori* infection in PPI-based triple therapy: A meta-analysis

**DOI:** 10.1097/MD.0000000000006489

**Published:** 2017-03-31

**Authors:** Meng Li, Taijie Li, Shihui Guo, Hongjie Liang, Dunke Jiang

**Affiliations:** a Department of Clinical Laboratory; b Department of Geriatric Gastroenterology, First Affiliated Hospital of Guangxi Medical University, Nanning, Guangxi, China.

**Keywords:** *helicobacter pylori*, MDR1, meta-analysis, polymorphism

## Abstract

**Background:**

Several studies have reported that multidrug resistance gene 1 (*MDR1*) *C3435T* polymorphism was associated with the rate of *Helicobacter pylori (H. pylori*) eradication in proton pump inhibitor (PPI)-based triple therapy. However, the conclusions were inconsistent. Therefore, this meta-analysis was conducted to evaluate the impact of *MDR1 C3435T* polymorphism on *H. pylori* eradication by PPI-based triple therapy.

**Methods:**

Seven eligible studies published up to August 2016 and including 1019 patients were identified by searching the Chinese Biomedical Literature database, Wan fang, PubMed, and the Web of Science electronic databases. Consequently, a meta-analysis was conducted with STATA software, using summary odds ratios (OR) and a 95% confidence interval (CI).

**Results:**

Overall, there was no significant difference between *MDR1 C3435T* polymorphism and the eradication rate of *H. pylori* in the entire genetic model, irrespective of the PPI used. Furthermore, in Asian populations, the TT genotype decreased *H. pylori* eradication (TT vs CT+CC: OR=0.411, 95% CI = 0.280–0.602, *P* = 0.000). In addition, a significantly low eradication rate was observed in a recessive model, in which either lansoprazole (TT vs CT+CC: OR = 0.305, 95% CI = 0.184–0.504, *P* = 0.000) or omeprazole (TT vs CT+CC: OR = 0.229, 95% CI = 0.069–0.763, *P* = 0.016) was taken, in a subanalysis of individual PPIs. In the analyses that were stratified by disease type, no significant difference was observed in the peptic ulcer group and the combined diseases subgroup.

**Conclusion:**

This meta-analysis indicated that the TT genotype of the *MDR1 C3435T* polymorphism decreased *H. pylori* eradication in Asian populations and was also associated with a low cure rate of *H. pylori* in patients taking lansoprazole- and omeprazole-based triple therapies. However, future studies using larger sample sizes are required.

## 1 Introduction

In recent years, increasing social pressure, an irregular diet, and dietary changes have increased the incidence of gastric ulcer. *Helicobacter pylori* (*H. pylori*), which is a chronic infectious pathogenic bacteria with a high worldwide infection rate, is the major risk factor for gastritis, peptic ulcer, mucosa-associated lymphoid tissue lymphoma, and gastric cancer.^[1,2]^ Moreover, the World Health Organization has recognized *H. pylori* as a class-1 carcinogenic factor.^[3]^ Hamajima et al^[4]^ reported that the risk of gastric cancer is 5 times higher in patients positive for *H. pylori* than in those without *H. pylori* infection in the Japanese population. Multiple studies have also documented that *H. pylori* eradication is essential in the treatment of gastric ulcer and in the prevention of ulcer recurrence, as well as in decreasing gastric cancer morbidity.

First-line triple therapy for *H. pylori* infection currently consists of a proton pump inhibitor (PPI) and 2 antibiotic agents, such as amoxicillin, metronidazole, or clarithromycin, for 1 week.^[5]^ However, it has been reported that the eradication rate of this treatment regimen is only 80% to 90%, and that it fails to eradicate *H. pylori* in the remaining approximately 20% of patients.^[6]^ Although a PPI can raise the intragastric pH, facilitate the stability and bioavailability of antibiotics, and eliminate the *H. pylori*,^[7]^ the failure of *H. pylori* eradication is associated with antibiotic resistance and patient tolerance. Some studies recently suggested that PPIs, such as omeprazole, lansoprazole, and pantoprazole, are substrates of P-glycoprotein (P-gp).^[8]^ The expression and function of P-gp is closely related to the absorption and metabolism of PPIs. P-gp is an ATP-dependent membrane-bound transporter that was first detected in tumor cells, and which is also expressed in normal tissues, such as the stomach, the small intestine, and the blood–brain barrier. This protein functions as an efflux pump that exports its substrates out of the cell, thereby affecting the absorption and elimination of numerous drugs.^[9,10]^

P-gp is encoded by the multidrug resistance gene 1 (*MDR1*). There is evidence to suggest that *MDR1* gene polymorphism could influence P-gp expression and then affect drug metabolism and pharmacokinetics. It was shown that *MDR1 C3435T* polymorphism in exon 26 is the most widely involved, which could affect the transport activity of P-gp, and that individual with the TT genotype had lower intestinal P-gp expression.^[11,12]^ Thus, it was assumed that *MDR1 C3435T* polymorphism might impact the pharmacokinetics and pharmacodynamics of PPIs, thereby affecting the *H. pylori* cure rate. Recent studies have pointed out that *MDR1 C3435T* polymorphism is related to the susceptibility of *H. pylori* infection-related gastritis and peptic ulcer.^[13,14]^ Studies also revealed that *MDR1 C3435T* polymorphism was associated with the eradication rates of *H. pylori* infection using PPI-based triple therapy, but the results were conflicting. The small sample size used in each study may have caused this deviation. In the present study, a meta-analysis was conducted to assess whether *MDR1 C3435T* polymorphism could influence the success or failure of *H. pylori* eradication by PPI-based triple therapy.

## 2 Materials and methods

### 2.1 Search strategy

A systematic literature search of the electronic databases of the Chinese Biomedical Literature database, Wan fang, PubMed, and the Web of Science for all publications up to August 2016 that evaluated the effect of *MDR1 C3435T* polymorphism on *H. pylori* eradication in PPI-based triple therapy was carried out. In addition, we also checked the reference lists of retrieved articles and reviews by hand-searching. The literature search was conducted without date and language restrictions, using the following Key words: “multidrug resistance 1 gene OR MDR1 OR P-glycoprotein OR ABCB1”; and *“Helicobacter pylori* OR *H. pylori* OR *Helicobacter* infection*”*; and “proton pump inhibitor(s) OR PPI(s) OR esomeprazole OR pantoprazole OR rabeprazole OR omeprazole OR lansoprazole.” The largest population or that; which used a complete study; was selected when more than 1 article used the same study population.

### 2.2 Inclusion and exclusion criteria

The following inclusion criteria had to be met in order for inclusion of a publication in the present study: PPI-based triple therapy for eradicating *H. pylori*, 7 to 14 days, first-line therapy, patients did not receive *H. pylori* eradication therapy before the study, patients were positive for *H. pylori* before treatment, the genotype of *MDR1 C3435T* polymorphism was provided. Exclusion criteria were as follows: reviews, or the *H. pylori* eradication therapy was irrelevant, duplicate data, insufficient information for data extraction.

### 2.3 Data extraction

All the data from each included paper were independently extracted by 2 reviewers, and discrepancies were resolved through discussion. The following variables were collated from each eligible study: year of publication, first author, country, ethnicity, genotyping method, *H. pylori* diagnosis methods, disease type, treatment regimen, triple therapy, PPI, eradication rates for the CC, CT, and TT genotypes, and effect of MDR1 genotype.

### 2.4 Statistical analysis

The odds ratio (OR), together with the corresponding 95% confidence interval (CI), was used to estimate the association between the *H. pylori* eradication rates and *MDR1 C3435T* polymorphism in an allele model (T vs C), a homozygous model (TT vs CC), a heterozygote model (TC vs CC), a dominant model (TT+TC vs CC), and a recessive model (TT vs TC+CC), respectively. In addition, subgroup analyses were conducted with regard to ethnicity, disease type, and individual PPIs. The studies that only estimated ulcer disease were included in the peptic ulcer subgroup,^[15–17]^ and the combined subgroup contained the studies that consisted of patients with gastric ulcer, duodenal ulcer, gastritis, and gastroesophageal reflux disease.^[18–21]^ The significance of the pooled OR was determined by a *Z* test and *P* < 0.05 was considered statistically significant.

A heterogeneity test was carried out for each included study, and these were evaluated using a *χ*^2^-based Q-statistic and an *I*^2^ statistic. When no significant heterogeneity was observed (*P* > 0.1 or *I*^2^≤50%), the fixed-effects model was used to assess the pooled OR. Otherwise, the random-effects model was used. Furthermore, the publication bias was estimated by Begg funnel plots and Egger test. No publication was observed when the *P* value of the Egger test was > 0.05. The present meta-analysis was performed using STATA Software (version 12.0; Stata Corporation, College Station, TX).

Meta-analysis is a systematic review based on previous studies and the ethical approval is not necessary.

## 3 Results

### 3.1 Study characteristics

**Figure 1:**
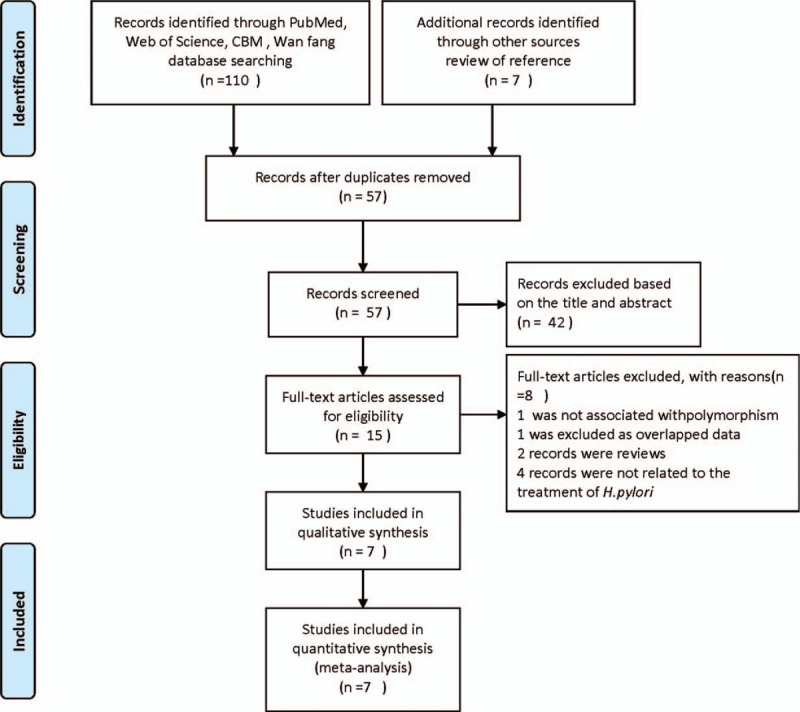
Flowchart illustrates the literature selection process of this meta-analysis.

The selection process of the eligible studies is shown in Fig. 1. Eight publications that assessed the association between *MDR1 C3435T* polymorphism and *H. pylori* eradication rate met the inclusion criteria. However, 1 article was excluded, after it was read in its entirety, as it used overlapping data.^[22]^ Therefore, 7 studies, which included 1019 patients, were used in our meta-analysis.^[15–21]^ Among these articles, 4 had been published in English, ^[15–17,21]^ and 3^[18–20]^ had been published in Chinese. Five studies had been conducted in Asia,^[16–20]^ and only 2 studies were Caucasian in origin.^[15,21]^*H. pylori* positivity was judged on the results of a ^13^C-urea breath test (^13^C-UBT), a ^14^C-urea breath test (^14^C-UBT), and a rapid urease test (RUT) before and after the triple treatment. Four reports ^[15–17,20]^ contained more than 1 disease, for example, gastric ulcer, gastritis, gastroesophageal reflux disease, functional dyspepsia, and duodenal ulcer, and these reports did not provide the *H. pylori* eradication rates for each disease. Therefore, in the subanalysis stratified by disease type, these 4 reports were included in the combined diseases subgroup, and the other 3 reports, which focused only on peptic ulcer or gastric ulcer, were included in the peptic ulcer subgroup.^[18,19,21]^ In these 7 studies, the numbers of omeprazole, pantoprazole, esomeprazole, and lansoprazole arms were 3, 3, 2, and 2, respectively. Two studies ^[18,20]^ contained omeprazole and esomeprazole-based triple therapy, and gave the *H. pylori* eradication rates for the CC, CT, and TT genotypes with each PPI. Therefore, they were considered 4 separate studies in the subanalysis of individual PPI treatments. Only 1 study, which was conducted by Gawronska-Szklarz et al^[15]^ showed a combined eradication rate with omeprazole together with pantoprazole-based triple therapy; this was included in the overall meta-analysis of all PPI-based therapies, but was excluded from the single arm subgroup analysis. The baseline characteristics of each original study are listed in Table 1.

**Table 1:**
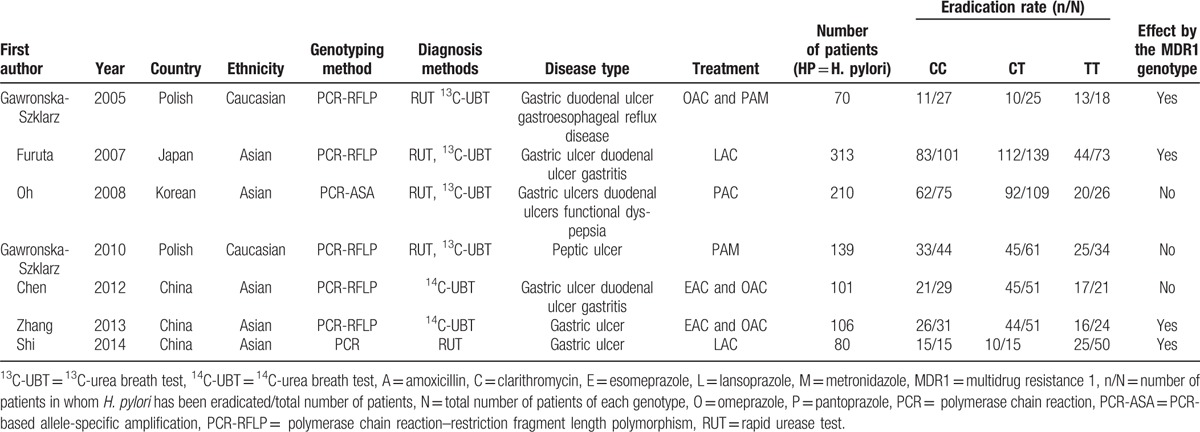
Baseline characteristics and the eradication rates of the articles included in this meta-analysis.

### 3.2 The effects of MDR1 C3435T polymorphism on the overall efficacy of all PPI-based triple therapies

Significant heterogeneity across all eligible studies was found in most of the genetic models, except for the CT versus CC comparison, so the random-effects model was used to assess pooled ORs. No significant difference was found between *MDR1 C3435T* polymorphism and the *H. pylori* eradication rate when all PPI-based triple therapies were combined in our initial analysis, irrespective of dose and antibiotics (Table 2).

**Table 2:**
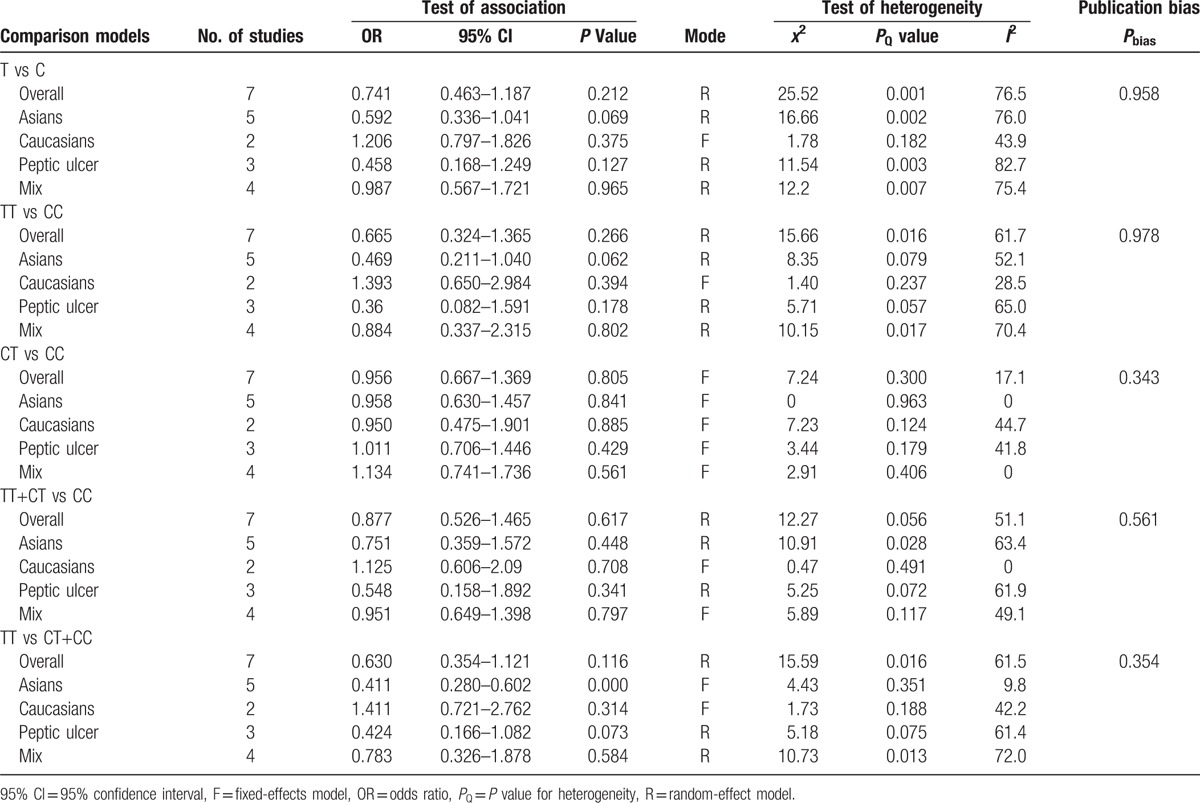
Meta-analysis of the effect of MDR1 C3435T genotype on eradication of *H. pylori*.

### 3.3 Subgroup analysis

**Figure 2:**
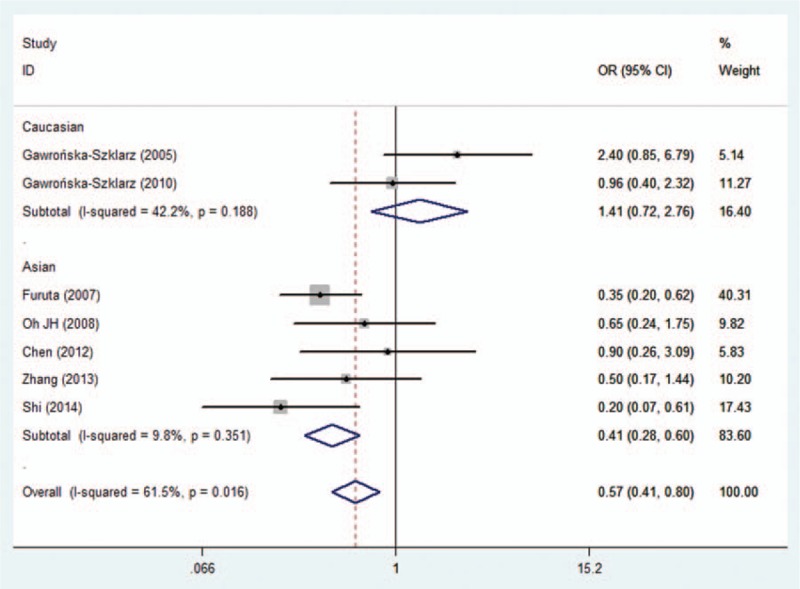
Forest plot for the association between MDR1 C3435T polymorphism and the eradication rate of *H. pylori* in the Asian population (TT vs CT+CC). *H. pylori* = *helicobacter pylori,* MDR1 = multidrug resistance 1.

In the analysis stratified by ethnicity, the results showed a statistically significant relationship between *MDR1 C3435T* TT genotype and *H. pylori* eradication rate (TT vs CT+CC: OR = 0.411, 95% CI = 0.280–0.602, *P* = 0.000, Table 2, Fig. 2) in Asian populations, and it implied that the patients with a TT genotype had a lower *H. pylori* eradication rate. However, no striking differences were observed in the other models (Table 2). With regard to the Caucasian populations, we did not find any significant relationships in any of the genetic models (Table 2). Furthermore, a subanalysis was carried out, stratifying by disease type. Statistical differences in *H. pylori* eradication rates for all *MDR1 C3435T* genotypes were not found in either the peptic ulcer or the combined digestive diseases subgroups (Table 2).

**Figure 3:**
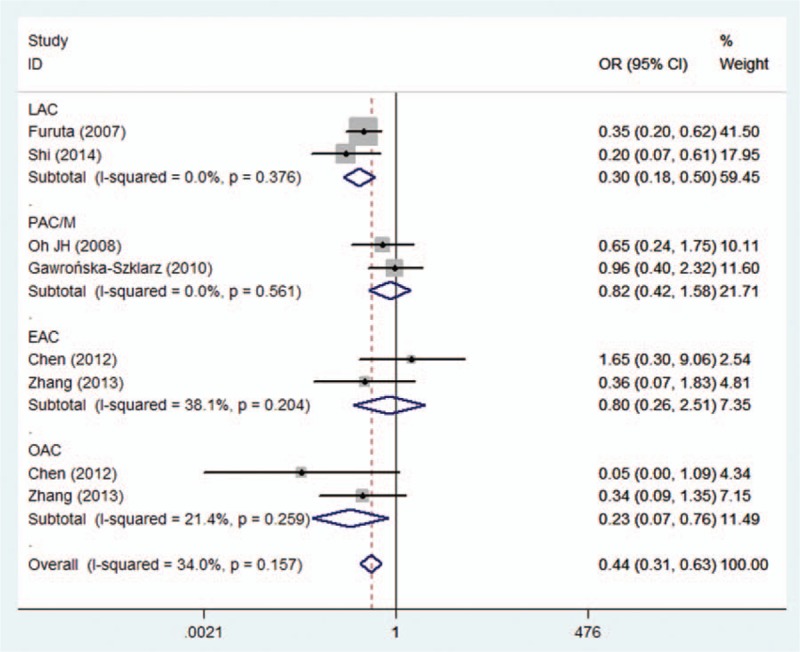
Forest plot of the MDR1 C3435T polymorphism and *H. pylori* eradication in the PPI-based therapies subgroup (TT vs CT+CC). *H. pylori* = *helicobacter pylori,* MDR1 = multidrug resistance 1, PPI = proton pump inhibitor.

In view of the possible differences in the effects of *MDR1 C3435T* polymorphism on *H. pylori* eradication rates with different PPIs, a subgroup analysis was carried out according to individual PPI-based triple therapies. Our subanalysis showed a significantly lower eradication rate with lansoprazole-based triple therapy in the recessive model (TT vs CT+CC: OR = 0.305, 95% CI = 0.184–0.504, *P* = 0.000, Table 3, Fig. 3). Similarly, a significant difference between *MDR1 C3435T* polymorphism and *H. pylori* eradication rates was observed in the recessive model for omeprazole-based triple therapy (TT vs CT+CC: OR = 0.229; 95% CI = 0.069–0.763, *P* = 0.016, Table 3, Fig. 3). However, there was no significant difference in *H. pylori* eradication rates between lansoprazole- and omeprazole-based triple therapies among the other genotypes. No significant differences in *H. pylori* eradication rates were found in any of the genotypes for pantoprazole or esomeprazole.

**Table 3:**
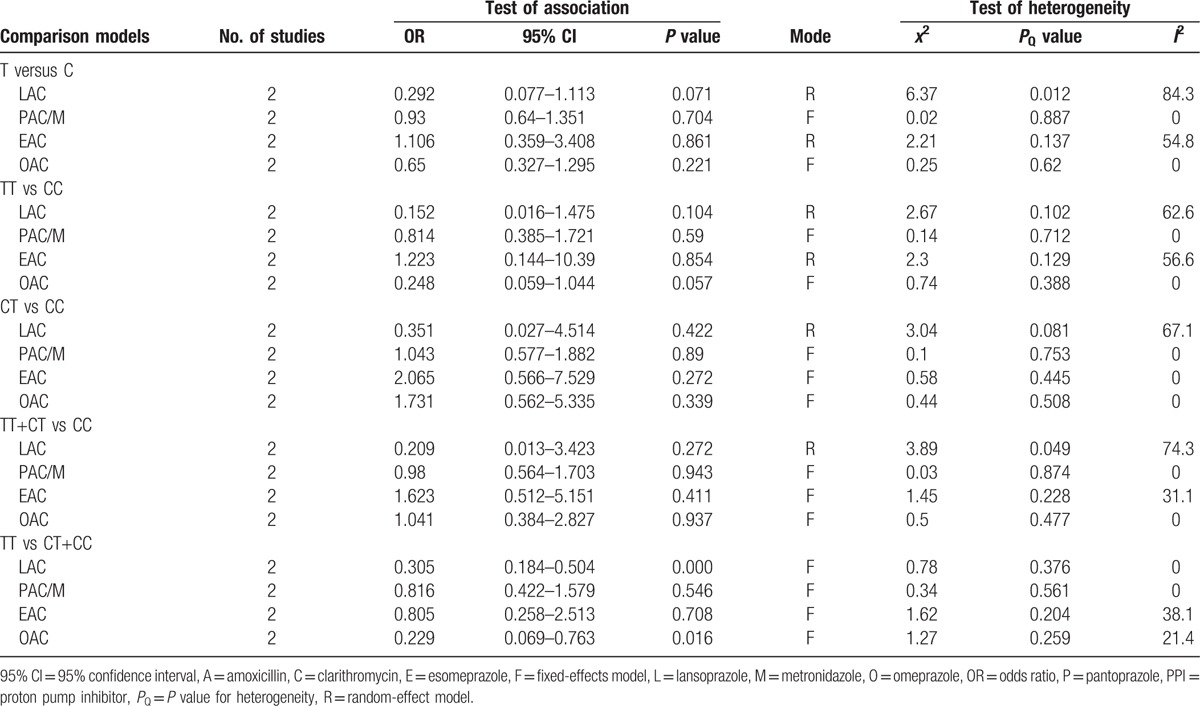
The effect of MDR1 C3435T genotype on eradication of *H. pylori* in the PPI-based therapies.

### 3.4 Publication bias

**Figure 4:**
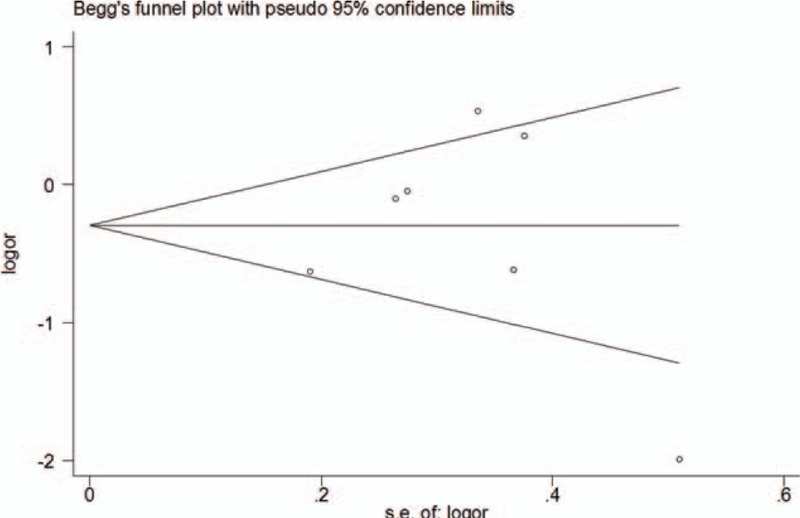
Funnel plot to assess the publication bias for T versus C allele genetic model in overall analysis.

The overall publication bias of our meta-analysis was estimated by Egger test and Begg funnel plots, and no statistically significant publication bias was found. When Egger test was conducted, no significant publication bias was detected for *MDR1 C3435T* polymorphism in any of the genetic models (*P* = 0.958 for T vs C; *P* = 0.978 for TT vs CC; *P* = 0.343 for CT vs CC; *P* = 0.561 for TT+CT vs CC; and *P* = 0.354 for TT vs CT+CC,). Similarly, the shape of the funnel plots was symmetrical for the *MDR1 C3435T* polymorphism allele model T vs C, overall (Fig. 4).

## 4 Discussion

The infection rate of *H. pylori* is the highest of all bacteria and nearly half of the general population is infected worldwide.^[23]^*H. pylori* cannot only cause mucosal damage, but may also lead to gastric acid secretion disorders; it has been closely associated with the incidence of gastritis, peptic ulcer diseases, and gastric cancer.^[24]^ Treatment to eradicate *H. pylori* is the key to effectively curing peptic ulcer, and reducing the incidence of gastric cancer.

It has been reported that *MDR1 C3435T* polymorphism could not only directly affect P-gp expression, but that it also affects the pharmacokinetics of numerous drugs. Previous research has shown that individuals who have the TT genotype have a higher plasma concentration of substrate drugs after oral administration than those with the CC or CT genotype.^[25]^ P-gp can bind to a drug that is its substrate, and pump the drug, or its metabolites, out of the cell, so as to reduce the intracellular drug concentration and lead to drug resistance.^[26]^ Previous studies have investigated the effects of *MDR1 C3435T* polymorphism on drug resistance and pharmacokinetics, for example, digoxin, cyclosporin A, and PPIs.^[27–29]^ PPIs are substrate for P-gp, and their absorption and metabolism are closely associated with the expression and function of P-gp.

To date, no final conclusion has yet been drawn regarding the effect of *MDR1 C3435T* polymorphism and the rate of *H. pylori* eradication in PPI-based triple therapy. Several critical factors could be responsible for the inconsistent results, such as sample size, differing ethnicity, PPI and antibiotics doses, single or combined gastrointestinal diseases, and the basic demographic characteristics of patients. Therefore, it is necessary to carry out a systematic evaluation of the effect of *MDR1 C3435T* polymorphism on the *H. pylori* eradication rate. To the best of our knowledge, the present meta-analysis, which included 7 studies consisting of 1019 *H. pylori*-positive cases, was the first study to investigate the effect of *MDR1 C3435T* polymorphism on the eradication rate of *H. pylori* infection by PPI-based triple therapy. We clearly showed that there was no significant difference in *H. pylori* eradication in any *MDR1 C3435T* polymorphism genotypes. Meta-analysis is a valuable tool for identifying disease genes, accumulating the published data from each previously published small study. Therefore, our results may be more reliable than those of each original study.

Several factors, such as ethnicity, disease types, treatment protocols, and the differing characteristics of patients (gender, age, smoking status, and alcohol consumption), might have contributed to the heterogeneity that existed in the overall populations across all of the included studies, and subgroup analysis was necessary. It has been reported that regional and racial differences exist in the distribution frequency of *MDR1 C3435T* polymorphisms. The distribution frequency of the TT genotype was higher in Asian populations than in Caucasian populations.^[30]^ Therefore, a subgroup meta-analysis stratified according to ethnicity was carried out to assess the influence of genetic background on the *H. pylori* eradication rate. We observed that this rate was reduced in the TT versus CT+CC recessive genetic model in the Asian subgroup, but not in the Caucasian populations. There were 5 studies, with a large combined sample size of 810 Asian populations, including those from Korea, Japan, and China, and this significantly improved the statistical power. However, with regard to Caucasian populations, there were only 2 (Polish) studies in the present meta-analysis, so a further, well-designed, study with a large sample size from a greater number of countries is necessary to validate our findings.

Drug efficacy was associated with disease severity and patient responses. The original studies estimated the relationship between *MDR1 C3435T* polymorphism and *H. pylori* eradication in a combination of digestive diseases, including gastritis, gastric ulcer, and duodenal ulcer, and the results were inconsistent. Previous studies have reported that patients with functional dyspepsia often respond poorly to eradication therapy, compared with individuals with peptic ulcer.^[31,32]^ In view of the complexity of the disease, a subanalysis was carried out, stratifying by disease type. Our results showed no significant association between *H. pylori* eradication rates and *MDR1 C3435T* polymorphisms in both the peptic ulcer and the combined digestive diseases subgroups. This suggested that combined disease might not have a noticeable effect on the final results.

In view of the possible differences in the molecular structure and metabolism of individual PPIs, a subgroup analysis was carried out to explore the effect of *MDR1 C3435T* polymorphism on *H. pylori* eradication rates for each PPI. We found that patients with the TT genotype had a lower *H. pylori* cure rate than those with the CC or CT genotype when lansoprazole- and omeprazole-based triple therapies were used. However, no such association was observed in the patients taking pantoprazole or esomeprazole. Omeprazole was the first-generation PPI; the molecular structure of lansoprazole is similar to that of omeprazole, and it is considered the second-generation PPI. Pantoprazole and esomeprazole are the new generation of PPIs, with 2 metabolic pathways, so they are less affected by *MDR1* polymorphism. Our results indicated that lansoprazole and omeprazole are affected by *MDR1* genotype status, and an increased dose and prolonged course of treatment with these 2 PPIs would be required to overcome the effect of *MDR1 C3435T* polymorphism on eradication rates. Given that our sample size might have been insufficient with regard to detecting differences in individual PPI treatments, a further study with large sample size is necessary.

Although our study was a systematic and comprehensive evaluation, some limitations should be noted. First, drug pharmacokinetics was influenced by numerous factors, and there were insufficient data to evaluate the influence of other elements. For example, mephenytoin hydroxylase (CYP2C19) was associated with *H. pylori* eradication by affecting the metabolism of PPI,^[33]^ and Furuta et al suggested that the total eradication rate decreased from 87% to 76%, as a result of the increased incidence of clarithromycin-resistant strains of *H. pylori*. ^[16,34]^ Second, only 2 studies of Caucasian subgroups, as well as each PPI subgroup, were included in our meta-analysis, and the limited samples might have weakened the ability to reach statistical significance. Finally, the total sample sizes were relatively small, which might have resulted in low statistical power. Despite the limitations, our study also has some advantages. It was the first systematic review to investigate the effect of *MDR1 C3435T* polymorphism on the eradication rate of *H. pylori* infection, and we conducted a detailed subgroup analysis stratified by individual PPI, ethnicity, and disease type. In addition, each eligible study met our inclusion criteria, and all of the patients were on first-line therapy. Furthermore, no significant publication bias was detected in all studies, which indicated that the results of our meta-analysis might be unbiased.

## 5 Conclusions

In conclusion, this meta-analysis indicated that TT homozygotes decreased *H. pylori* eradication in Asians. Furthermore, the efficacy of lansoprazole- and omeprazole-based triple therapies was dependent on *MDR1* genotype status, but *MDR1* genotypes did not influence the *H. pylori* cure rate in triple therapy with pantoprazole or with esomeprazole. Considering the limitations of this meta-analysis, large sample studies, including other populations, are required to validate our results.
